# Identification of Dysbetalipoproteinemia by an Enhanced Sampson-NIH Equation for Very Low-Density Lipoprotein-Cholesterol

**DOI:** 10.3389/fgene.2022.935257

**Published:** 2022-07-14

**Authors:** Maureen Sampson, Anna Wolska, Jeff W. Meeusen, Leslie J. Donato, Allan S. Jaffe, Alan T. Remaley

**Affiliations:** ^1^ Department of Laboratory Medicine, Clinical Center, National Institutes of Health, Bethesda, MD, United States; ^2^ Lipoprotein Metabolism Laboratory, Translational Vascular Medicine Branch, National Heart, Lung, and Blood Institute, National Institutes of Health, Bethesda, MD, United States; ^3^ Department of Laboratory Medicine and Pathology, Mayo Clinic, Rochester, MN, United States; ^4^ Department of Cardiology, Mayo Clinic, Rochester, MN, United States

**Keywords:** ASCVD, cholesterol, dysbetalipoproteinemia, equation, VLDL

## Abstract

Dysbetalipoproteinemia (hyperlipoproteinemia type III, HLP3) is a genetic disorder that results in the accumulation of cholesterol on highly atherogenic remnant particles. Traditionally, the diagnosis of HLP3 depended upon lipoprotein gel electrophoresis or density gradient ultracentrifugation. Because these two methods are not performed by most clinical laboratories, we describe here two new equations for estimating the cholesterol content of VLDL (VLDL-C), which can then be used for the diagnosis of HLP3. Using results from the beta-quantification (BQ) reference method on a large cohort of dyslipidemic patients (N = 24,713), we identified 115 patients with HLP3 based on having a VLDL-C to plasma TG ratio greater than 0.3 and plasma TG between 150 and 1,000 mg/dl. Next, we developed two new methods for identifying HLP3 and compared them to BQ and a previously described dual lipid apoB ratio method. The first method uses results from the standard lipid panel and the Sampson-NIH equation 1 for estimating VLDL-C (***S***-VLDL-C), which is then divided by plasma TG to calculate the VLDL-C/TG ratio. The second method is similar, but the Sampson-NIH equation 1 is modified or enhanced (***eS***-VLDL-C) by including apoB as an independent variable for predicting VLDL-C. At a cut-point of 0.194, the first method showed a modest ability for identifying HLP3 (sensitivity = 73.9%; specificity = 82.6%; and area under the curve (AUC) = 0.8685) but was comparable to the existing dual lipid apoB ratio method. The second method based on ***eS***-VLDL-C showed much better sensitivity (96.5%) and specificity (94.5%) at a cut-point of 0.209. It also had an excellent AUC score of 0.9912 and was superior to the two other methods in test classification. In summary, we describe two new methods for the diagnosis of HLP3. The first one just utilizes the results of the standard lipid panel and the Sampson-NIH equation 1 for estimating (VLDL-C) (***S***-VLDL-C) and can potentially be used as a screening test. The second method (***eS***-VLDL-C), in which the Sampson-NIH equation 1 is modified to include apoB, is nearly as accurate as the BQ reference method. Because apoB is widely available at most clinical laboratories, the second method should improve both the accessibility and the accuracy of the HLP3 diagnosis.

## Introduction

Dysbetalipoproteinemia, which is often also called hyperlipoproteinemia Type III (HLP3) or broad beta (β) disease, is a highly atherogenic genetic disorder of lipoprotein metabolism (Hopkins et al., 2014). HLP3 patients have a marked increased risk of both coronary artery disease (CAD) and peripheral vascular disease (Mahley et al., 1999; Yuan et al., 2007). The prevalence of HLP3 in the U.S. was calculated to be approximately 2% based on a cross-sectional analysis of U.S. adults from the National Heart and Nutrition Examination Survey and the Very Large Database of Lipids (Pallazola et al., 2019) but is likely more prevalent in patients seen at lipid clinics with mixed dyslipidemias. It is usually characterized by an almost equal increase in total plasma cholesterol (TC) and triglycerides (TG), although the elevation of these two lipids is relatively modest compared to some other types of dyslipidemias. The main metabolic abnormality in HLP3 is the accumulation of abnormal cholesterol-enriched β-migrating very low-density lipoproteins (ß-VLDL) due to impaired TG lipolysis and plasma clearance (Mahley et al., 1999). Patients with HLP3 are often first diagnosed in middle adulthood by laboratory testing or by the presence of either striate palmer xanthomas, which are considered pathognomonic for this disorder, or by the presence of tubero-eruptive xanthomas on other body surfaces, which occur in other types of dyslipidemias (Durrington, 2007).

The apolipoprotein E2 (apoE) isoform, which differs from the most common apoE3 isoform by a single amino acid substitution (Cys150→Arg), is associated with HLP3 (Mahley et al., 1999). Homozygosity of apoE2 results in reduced binding of VLDL particles to the low-density lipoprotein-receptor (LDLR) and related lipoprotein receptors, but not all homozygous patients for apoE2 have clinically manifested disease, and thus, secondary contributory factors are often also involved (Koopal et al., 2017). Dominantly inherited forms of HLP3 have also been reported for other single amino acid substitutions in apoE (Mahley et al., 1999) (Arg136→Ser/Cys; Arg142→Cys/Leu; Arg145→Cys; Lys146→Gln/Glu; Lys146→Asn; Arg147→Trp) or from the insertion of a tandem repeat of amino acids (apoE-Leiden; residues 121–127). Secondary factors that can contribute to the formation of the HLP3 phenotype in patients with predisposing apoE variants include obesity, diabetes, and hypothyroidism (Durrington, 2007). There is no specific therapy for HLP3, but making this diagnosis is prognostically useful because these patients are at a greater risk for atherosclerotic cardiovascular diseases (ASCVD) than would be estimated from their plasma lipid profile (Hopkins et al., 2005).

Results from the standard lipid panel, TC, TG, low-density lipoprotein-cholesterol (LDL-C), and high-density lipoprotein-cholesterol (HDL-C) cannot be used to identify HLP3 because of an overlap with other dyslipidemias (Sniderman et al., 2018). The gold standard method for identifying HLP3 is the demonstration of the presence of the ß-VLDL band by agarose gel electrophoresis. Alternatively, increased levels of cholesterol in VLDL (VLDL-C) isolated after density gradient ultracentrifugation have also long been used for identifying this disorder (Fredrickson et al., 1967). A VLDL-C/plasma TG ratio of 0.3 or more (if lipids are expressed in mg/dL) when plasma TG is between 150 and 1,000 mg/dl is the criteria most often used when the diagnosis is based on ultracentrifugation (Fredrickson et al., 1967; Fredrickson et al., 1975). Because neither agarose gel electrophoresis nor ultracentrifugation is widely used by routine clinical laboratories, the ratio of plasma TC and TGs to apolipoprotein B (apoB), the main structural protein component of VLDL, has been used to screen for HLP3 (Sniderman et al., 2007).

In 2020, we described a new and more accurate equation for estimating LDL-C (Sampson-NIH equation) based on the results of the standard lipid panel (Sampson et al., 2020). This equation consists of two components: equation 1 for estimating VLDL-C, which we call here the original Sampson VLDL-C equation (***S***-VLDL-C), and the other was Sampson-NIH equation 2 for LDL-C. The main improvement in more accurately estimating LDL-C was the more accurate estimate of VLDL-C. ***S***-VLDL-C was designed to match the level of VLDL-C found in hypertriglyceridemic (HTG) patients when measured by the reference ultracentrifugation method, beta-quantification (BQ). The ***S***-VLDL-C equation was more accurate than the Friedewald formula for VLDL-C (***F***-VLDL-C), which assumes a constant VLDL-C/TG ratio of 0.2 (Friedewald et al., 1972), or the Martin method, which uses 180 empirically derived VLDL-C/TG ratios ranging from 0.3 to 0.08 (Martin et al., 2013).

In this study, we first examined whether the improved estimate of VLDL-C by the ***S***-VLDL-C equation could be used for identifying HLP3, and we found that it can potentially be used as a screening test for HLP3. Next, we developed the new enhanced Sampson equation for VLDL-C (***eS***-VLDL-C), which is a modified ***S***-VLDL-C equation with added apoB as an independent variable. The ***eS***-VLDL-C equation had the best specificity and sensitivity for identifying HLP3 compared to ***S***-VLDL-C and ***F***-VLDL-C.

## Methods

Deidentified lipid test results were obtained from the cardiovascular laboratory medicine program at Mayo Clinic in Rochester, MN. All results from lipoprotein metabolism profiling (which includes lipoprotein electrophoresis, apoB measurement, and the BQ reference method) ordered for clinical management between 2011 and 2021 were included (N = 24,713).

The BQ reference method was performed by a combination of ultracentrifugation and LDL precipitation as previously described (Meeusen et al., 2014; Meeusen et al., 2015). Cholesterol and nonglycerol blanked TG were measured by enzymatic methods on a Cobas 501 instrument (Roche Diagnostics, IN). Samples with detectable Lipoprotein-X on agarose gel electrophoresis (N = 126) and/or TG >3,000 mg/dl (N = 38) or TC >1,500 mg/dl (N = 3) were excluded from analysis. VLDL-C was calculated by subtracting cholesterol in the infranatant (d <1.006 g/ml), which contains LDL and HDL, from TC in plasma. ApoB was measured by the immunoturbidometric method, using a Cobas c501 analyzer (Roche Diagnostics, IN). HLP3 was defined as having a VLDL-C to plasma TG ratio greater than 0.3 and plasma TG between 150 and 1,000 mg/dl (Fredrickson et al., 1975) as determined from the BQ analysis.

The BQ dataset was randomly divided into a training dataset (N = 12,141) to first develop by a regression analysis the newly described equations, which were then validated in the other half of the data (N = 12,405). The least-square analysis for developing the new ***eS***-VLDL-C equation was done first by calculating values for the terms in the ***S***-VLDL-C equation in Excel, using the results of the standard lipid panel and then adding separate terms for apoB and an interaction term between apoB and TG. Stepwise multiple regression for predicting VLDL-C as measured by BQ was then performed with JMP software for calculating the coefficient values for each term of the ***eS***-VLDL-C equation. A similar approach was used to develop the HLP3-specific equations for predicting the cholesterol and TG content of VLDL, but only those patients identified as having HLP3 by BQ were used during the regression analysis. The receiver operating characteristic (ROC) analysis was also done with JMP software, and the optimum cut-point was calculated based on the value that gave the greatest sum of sensitivity plus specificity for identifying HLP3 when compared to the classification by the BQ analysis. Percent concordance with the BQ method for identifying HLP3 was done by calculating the percent of correctly identified patients (true positives + true negatives) out of the total number of test classifications (true positives + true negatives + false positives + false negatives). VLDL-C and LDL-C were also calculated by Sampson equations 1 and 2, respectively, as previously described (Sampson et al., 2020) using Excel. Mean lipid values were compared between groups by nonpaired *t*-test. Excel spreadsheets for performing all the calculations, including the new equations described here, can be freely downloaded at https://figshare.com/articles/software/Sampson_enhanced_VLDLC_phenotype_calculator/19666347.

## Results

Lipid values as determined by the BQ reference method and demographic characteristics of patients in the cohort are shown in Table 1. Based on these results, approximately 0.5% of patients were classified as having HLP3 by having a VLDL-C/TG ratio ≥0.3 and a plasma TG between 150 and 1,000 mg/dL. As expected, patients classified as HLP3 had modest increases in plasma TC, non-HDL-C, and TG. They also had an increase in the mean level of apoB compared to the non-HLP3 patients, but the relative increase in apoB was less than for the changes observed in plasma lipids. Although VLDL-TG was also increased in HLP3, there was a much larger relative increase in VLDL-C, leading to a mean VLDL-C/TG ratio of 0.4, which was more than two times the mean value observed in the non-HLP3 group. We did not observe any significant difference in the sex distribution and only a small difference in the mean age between the two groups.

**TABLE 1:** Lipid test values and demographics of the patient cohort.

	Non-HLP3 (N = 24,431)			HLP3 (N = 115)			
Test	Mean (IQR)	Min	Max	Mean (IQR)	Min	Max	*p* value
TC	194 (157–225)	27	672	340 (243–401)	171	811	<0.0001
Non-HDL-C	147 (111–175)	12	657	301 (208–372)	132	777	<0.0001
TG	164 (83–179)	5	2,931	375 (212–512)	152	811	<0.0001
ApoB	99 (78–117)	5	377	118 (79–144)	50	401	<0.0001
HDL-C	47 (37–56)	2	201	40 (31–44)	16	90	<0.0001
HDL-TG	18 (11–23)	3	359	22 (12–28)	3	120	0.0022
LDL-C	121 (90–146)	7	593	149 (93–169)	53	628	<0.0001
LDL-TG	45 (30–51)	0	577	87 (53–103)	31	260	<0.0001
VLDL-C	26 (13–30)	0	573	152 (85–203)	46	512	<0.0001
VLDL-TG	101 (34–110)	0	2,674	267 (134–335)	35	678	<0.0001
VLDL-C/TG	0.16 (0.13–0.19)	0.00	0.69	0.4 (0.32–0.44)	0.30	0.69	<0.0001
Age	55 (45–66)	18	90	52 (44–60)	23	90	0.0244
%male	50	—	—	50	—	—	0.4428

**FIGURE 1:**
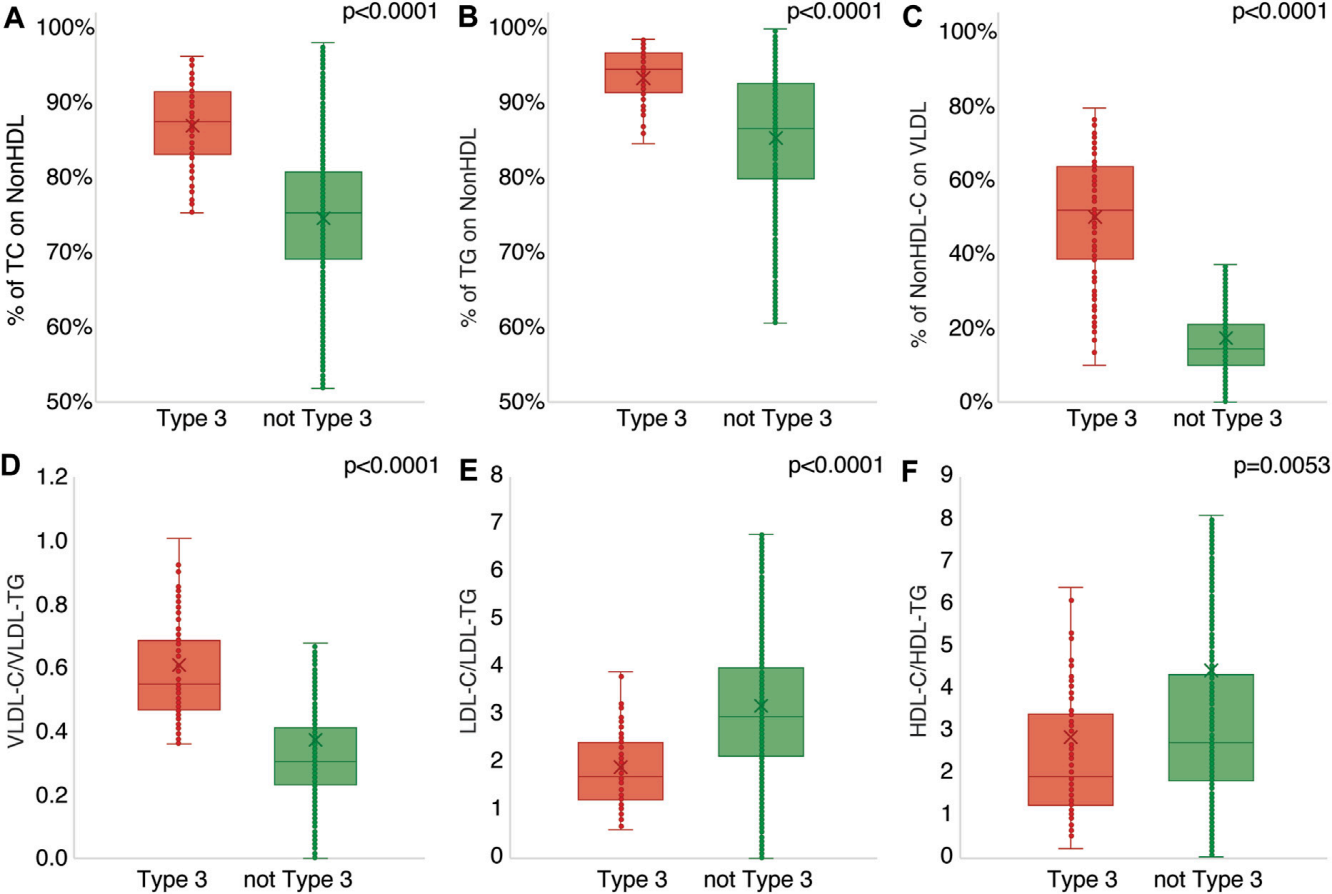
Comparison of lipid ratios between HLP3 and non-HLP3 patients. Specimens determined to have HLP3 (N = 115, red) by BQ were compared against those without HLP3 (N = 24,431, green) for the % of TC on non-HDL **(A)**, % of TG on non-HDL **(B)**, % of non-HDL-C on BQ VLDL **(C)**, the ratios of VLDL-C/VLDL-TG **(D)**, LDL-C/LDL-TG **(E)**, and HDL-C/HDL-TG **(F)**.

We also observed enrichment of TG in both HDL and LDL in HLP3 patients (Table 1), suggesting an abnormal lipid composition for these lipoprotein particles too. This was further analyzed in Figure 1 by examining the ratio of cholesterol and TG in the major lipoprotein fractions. A much greater fraction of plasma TC, almost 90% on average, was present in the non-HDL fraction for HLP3 patients (Figure 1A). This was due to the lower level of HDL-C in these patients and the enrichment of cholesterol on VLDL (Table 1). The percent of TG found in non-HDL was also higher for HLP3 than non-HLP3 (Figure 1B), but the difference was less than what was observed for cholesterol (Figure 1A). Unlike the non-HLP3 group in which most of the cholesterol that makes up non-HDL-C is in LDL, we found that approximately half of the cholesterol on nonHDL particles is found in VLDL in HLP3 patients (Figure 1C). As previously reported, this is a consequence of delayed plasma clearance of VLDL and the replacement of TG in VLDL for cholesteryl esters (CE) by Cholesteryl Ester Transfer Protein (CETP) mediated lipid exchange (Barter et al., 2003), which results in the increased VLDL-C/VLDL-TG ratio in HLP3 (Figure 1D). CETP-mediated lipid exchange likely also accounts for the observed enrichment of TG over cholesterol in both LDL and HDL (Figures 1E,F) because when CETP removes TG from VLDL in exchange for CE, it causes the TG-enrichment of LDL and HDL (Barter et al., 2003).

In addition to the standard lipid panel, the only other routine lipid-related diagnostic test that could potentially improve VLDL-C estimation is apoB, which is present as a single copy per VLDL particle (Elovson et al., 1988). Using the same terms as in the original Sampson-NIH equation 1 for VLDL-C, called here ***S***-VLDL-C (Sampson et al., 2020), and adding apoB and an interaction term between apoB and TG, we developed by the regression analysis the following enhanced Sampson VLDL-C equation (***eS***-VLDL-C):

$$\mathrm{eS‐VLDL‐C}=\frac{\mathrm{non‐HDL}‐C}{3.81}-\;\frac{\mathrm{HDL}‐C}{8.93}+\;\frac{\mathrm{TG}}{7.73}+\;\frac{\left(\mathrm{non‐HDL}‐C\times \mathrm{TG}\right)}{2050}-\;\frac{TG^{2}}{13300}-\;\frac{\mathrm{ApoB}}{2.49}-\;\frac{\left(\mathrm{ApoB}\times \mathrm{TG}\right)}{3550}+7.46.$$

**FIGURE 2:**
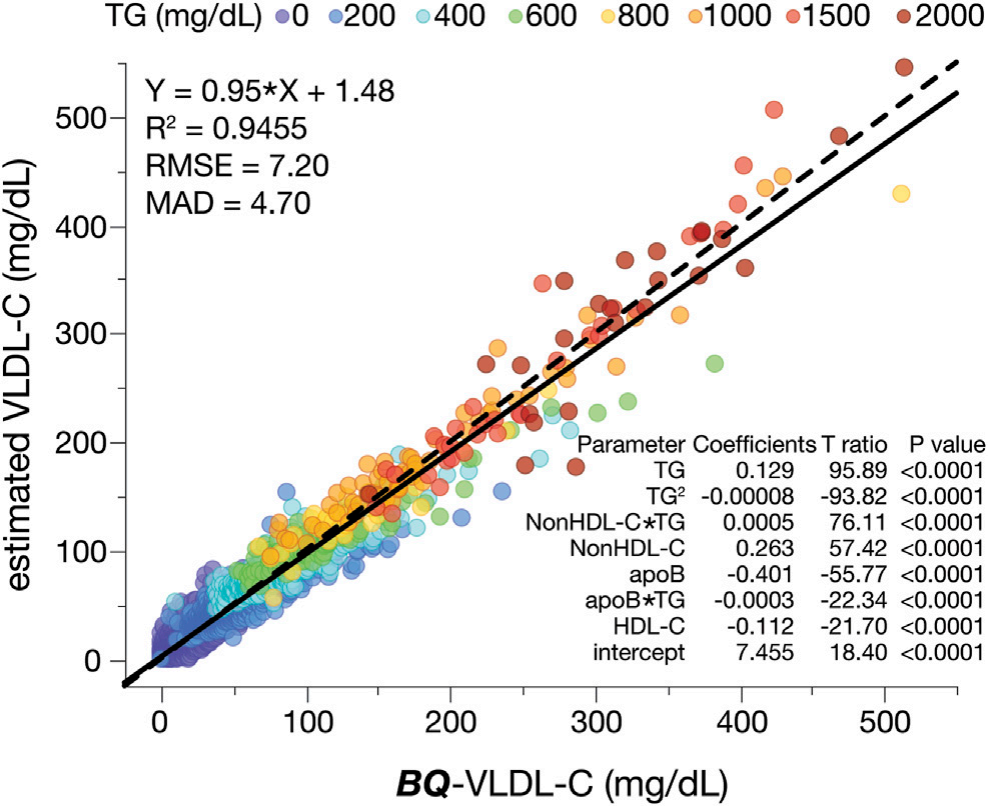
Development of the enhanced VLDL-C equation (*eS*-VLDL-C). The training dataset (N = 12,141) was used in multiple regression analysis with the indicated input variables. The solid line is the linear fit for indicated regression equation. A dashed line is the line of identity. Results are color-coded by TG levels with the value in the legend indicating the start of each interval.

Based on the mean absolute difference (MAD) and other quantitative metrics of accuracy (slope, intercept, correlation coefficient (R^2^), and root mean square error (RMSE)), the ***eS***-VLDL-C equation appeared to yield relatively accurate results, which had a broad range of VLDL-C (0–514 mg/dl) and TG values (5–2,853 mg/dl) in the training dataset (Figure 2).

**FIGURE 3:**
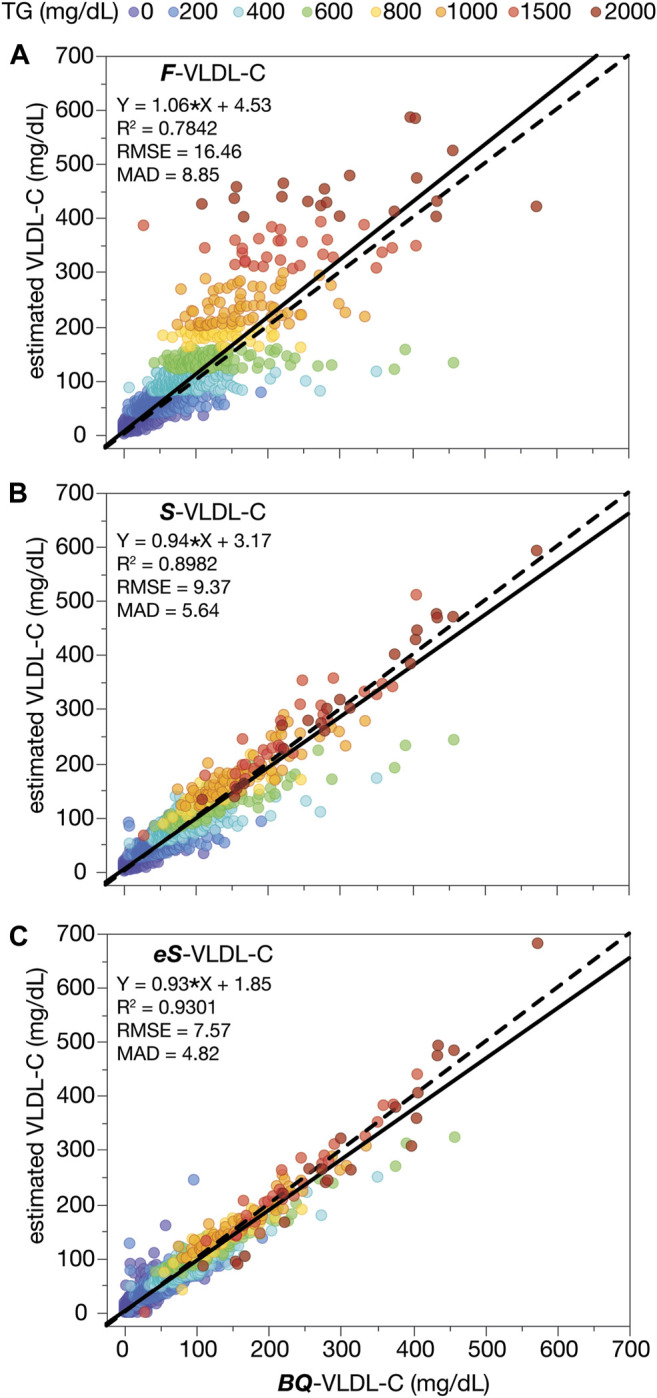
VLDL-C by calculation versus β-quantification. VLDL-C was calculated from the results of a standard lipid panel in the validation dataset (N = 12,405) by the Friedewald VLDL-C formula (*F*-VLDL-C) **(A)**, the Sampson VLDL-C equation (*S-*VLDL-C) **(B)**, and the enhanced Sampson VLDL-C equation (*eS*-VLDL-C) **(C)** and plotted against VLDL-C as measured by BQ. Solid lines are the linear fit for indicated regression equations. Dashed lines are the line of identity. Results are color-coded by TG levels with the value in the legend indicating the start of each interval.

Next, we evaluated the accuracy of the new ***eS***-VLDL-C equation, the ***S***-VLDL-C equation, and the Friedewald equation (TG/5) for VLDL-C (***F***-VLDL-C) in the validation dataset when compared to the BQ reference method (Figure 3). Based on MAD and other metrics of accuracy shown in the three plots, the ***eS***-VLDL-C equation (Figure 3C) showed the greatest accuracy followed by the original ***S***-VLDL-C equation (Figure 3B) and then the ***F***-VLDL-C equation (Figure 3A). As has been well described (Sampson et al., 2020), the Friedewald equation shows a positive bias for VLDL-C with increasing TG. In addition, the Friedewald equation for VLDL-C shows a marked increase in its variance for high TG samples, making it unreliable when TG is greater than 400 mg/dl. This problem occurs to a lesser degree with the ***S***-VLDL-C equation and an even lesser degree with the ***eS***-VLDL-C equation that includes apoB for estimating VLDL-C.

**FIGURE 4:**
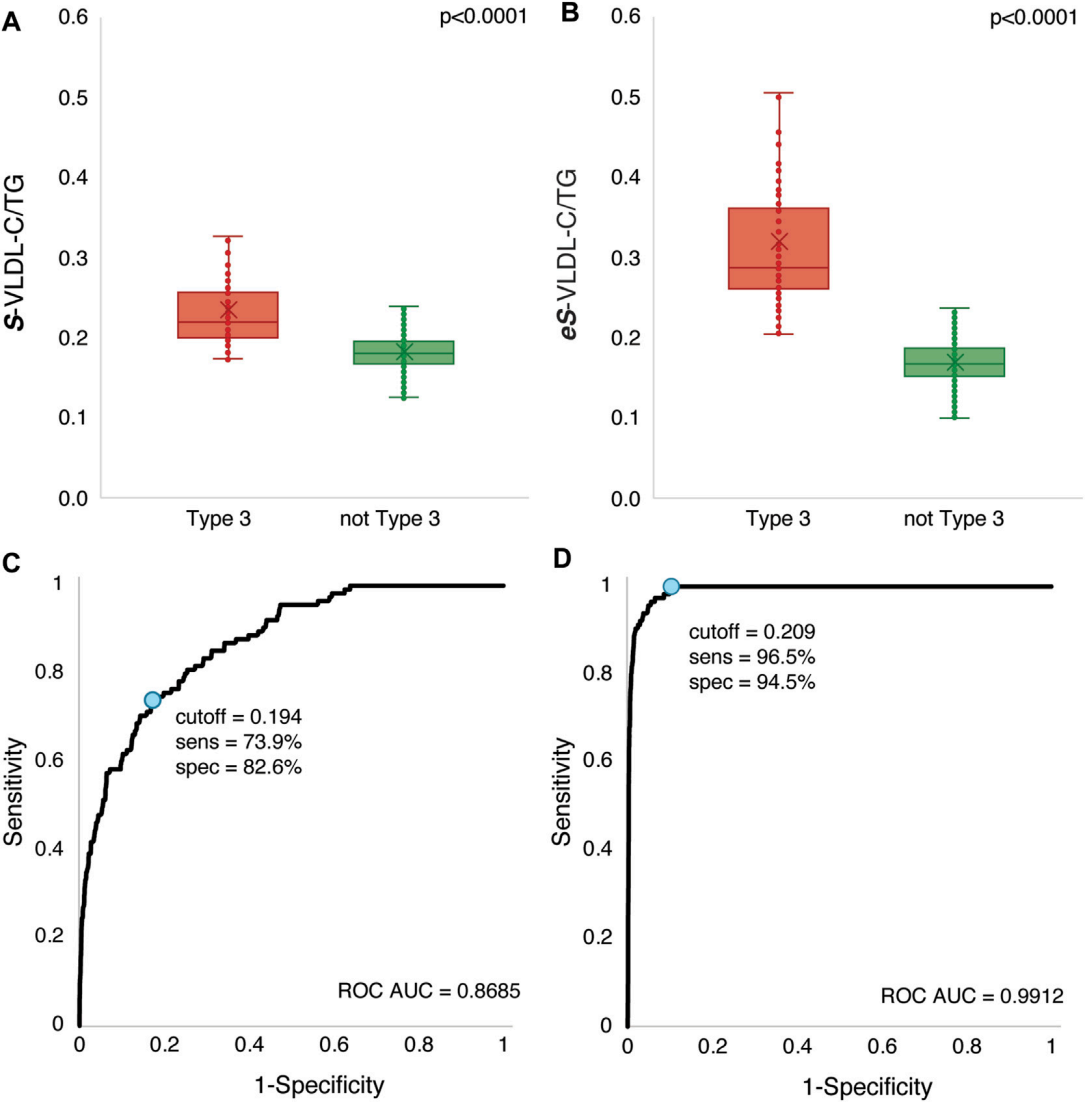
VLDL-C/TG ratio comparison in HLP3 and non-HLP3 patients. VLDL-C was calculated by the Sampson VLDL-C/TG (*S*-VLDL-C/T) **(A)** and enhanced Sampson VLDL-C/TG (*eS*-VLDL-C/T) **(B)** equations in the validation dataset (N = 12,405) from the results of a standard lipid panel. HLP3 was defined as VLDL-C/TG > 0.3 mg/dl and TG between 150–1,000 mg/dl using the BQ results. The ROC curves are plotted for the Sampson VLDL-C/TG (*S*-VLDL-C/T) **(C)** and enhanced Sampson VLDL-C/TG (*eS*-VLDL-C/T) **(D)** equations. The optimal cut-point (blue circle) and its corresponding sensitivity and specificity are indicated for each equation.

In Figure 4, the VLDL-C to plasma TG ratio (VLDL-C/TG) was calculated by the ***S***-VLDL-C equation and the new ***eS***-VLDL-C equation and compared to results obtained by the BQ reference method. Only a small percent of patients (11%) identified as having HLP3 by BQ had a VLDL-C/TG ratio greater than 0.3 when VLDL-C was calculated by the ***S***-VLDL-C equation (Figure 4A). Those patients classified as non-HLP3 by the BQ reference method did, however, have a much lower VLDL-C/TG ratio than the HLP3 group. In contrast, approximately 41% of HLP3 patients as determined by BQ did have a VLDL-C/TG ratio greater than 0.3 when calculated by the ***eS***-VLDL-C equation (Figure 4B).

Because HLP3 patients were only a small minority of the total number of patients that we used to develop our regression equations for estimating VLDL-C, it is not unexpected that the regression equations would underestimate VLDL-C given that the mean VLDL-C level for the HLP3 group is almost 5 times higher than the non-HLP3 group (Table 1). We, therefore, investigated by the ROC analysis whether a different cut-point for the VLDL-C/TG ratio could better discriminate between the two groups. At a cut-point of 0.194, the ***S***-VLDL-C equation did show a modest ability to identify HLP3, with a sensitivity of 73.9% and a specificity of 82.6% (Figure 4C). It also showed a moderately good AUC score, an overall metric of test classification, of 0.8685. At a cut-point of 0.209, the ***eS***-VLDL-C, however, showed much better sensitivity (96.5%) and specificity (94.5%) and an excellent AUC score of 0.9912 (Figure 4D).

**FIGURE 5:**
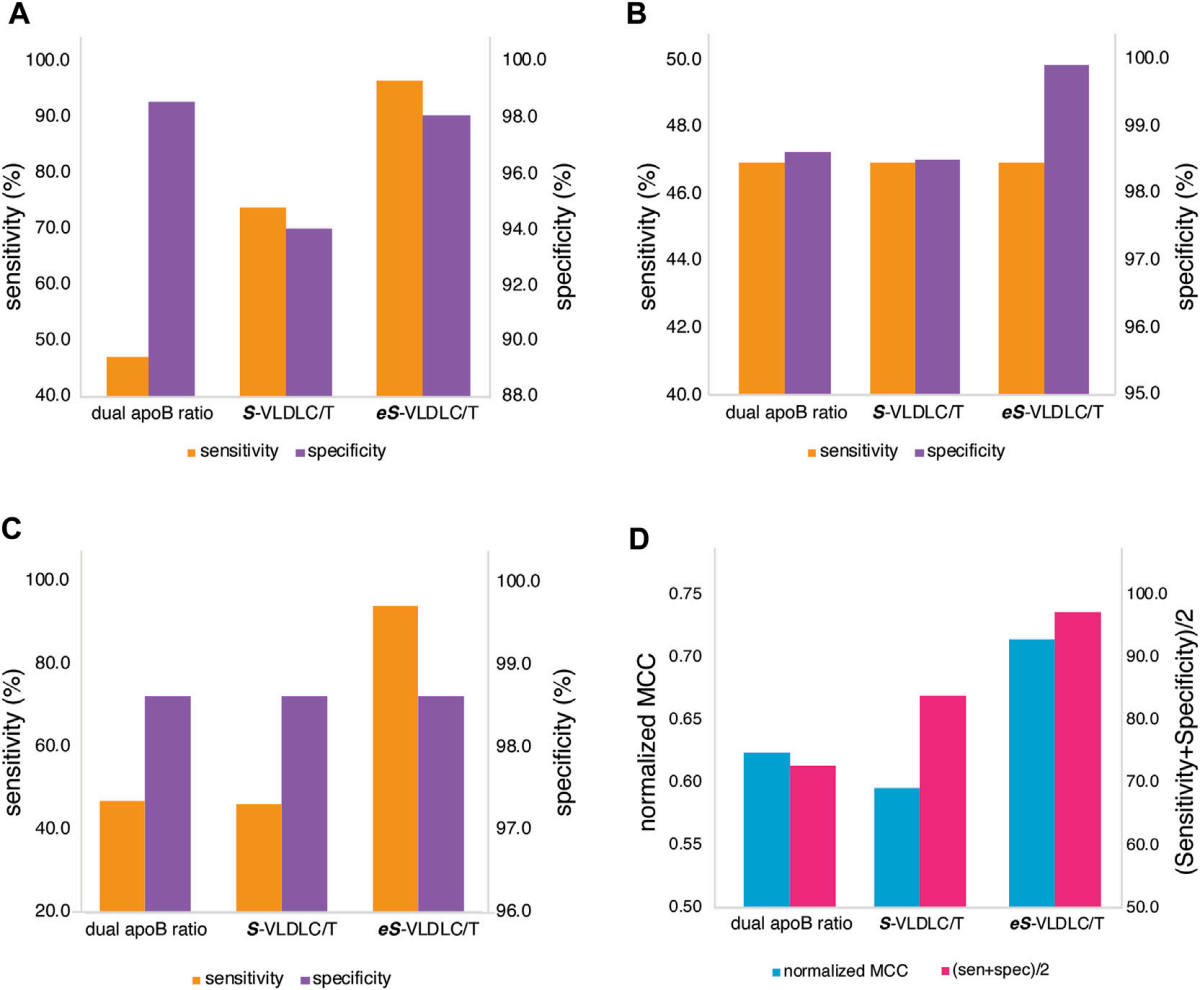
Comparison of test accuracy for HLP3 classification. Classification accuracy in the validation dataset (N = 12,405) by sensitivity (orange) and specificity (purple) for the dual apoB ratio method, the Sampson VLDL-C/TG (*S*-VLDL-C/T), and enhanced Sampson VLDL-C/TG (*eS*-VLDL-C/T) methods are shown for the optimal cut-points **(A)**, after adjustment to match the dual apoB ratio sensitivity **(B)**, and after adjustment to match the dual apoB ratio specificity **(C)**. The normalized MCC score (blue) and the average of sensitivity and specificity (sen + spec/2; pink) are shown for the optimal cut-points **(D)**.

Next, we compared the ***S***-VLDL-C and ***eS***-VLDL-C equations to the previously described dual lipid apoB ratio method (Sniderman et al., 2007; Sniderman et al., 2018), which requires both TC/apoB≥6.2 and TG/apoB≤10 ratios (when units are in mmol/L) for identifying HLP3 (Figure 5). When used at the near-optimal cut-point shown in Figure 4, the ***S***-VLDL-C equation was much more sensitive than the dual lipid apoB ratio test but much less specific (Figure 5A). The ***eS***-VLDL-C equation at its optimal cut-point was more sensitive than the dual apoB ratio method but had a similar specificity. The ***eS***-VLDL-C equation was both more sensitive and specific than the ***S***-VLDL-C equation (Figure 5A) and had an overall concordance with the BQ method of 94.6%. Next, we changed the cut-point of the ***S***-VLDL-C and ***eS***-VLDL-C equations to match either the sensitivity (Figure 5B) or specificity (Figure 5C) of the dual lipid apoB ratio method. Based on this analysis, the ***S***-VLDL-C equation was similar in diagnostic test performance to the dual lipid apoB ratio method for identifying HLP3, and the ***eS***-VLDL-C equation appeared to show overall the best sensitivity and specificity compared to the two other methods. Similar conclusions were found on the relative diagnostic value of the three methods by giving equal value to sensitivity and specificity (Sen + Spec/2) and by using the normalized Matthews correlation coefficient (MCC) index (Figure 5D), which adjusts for the prevalence of disease (Chicco et al., 2021).

**FIGURE 6:**
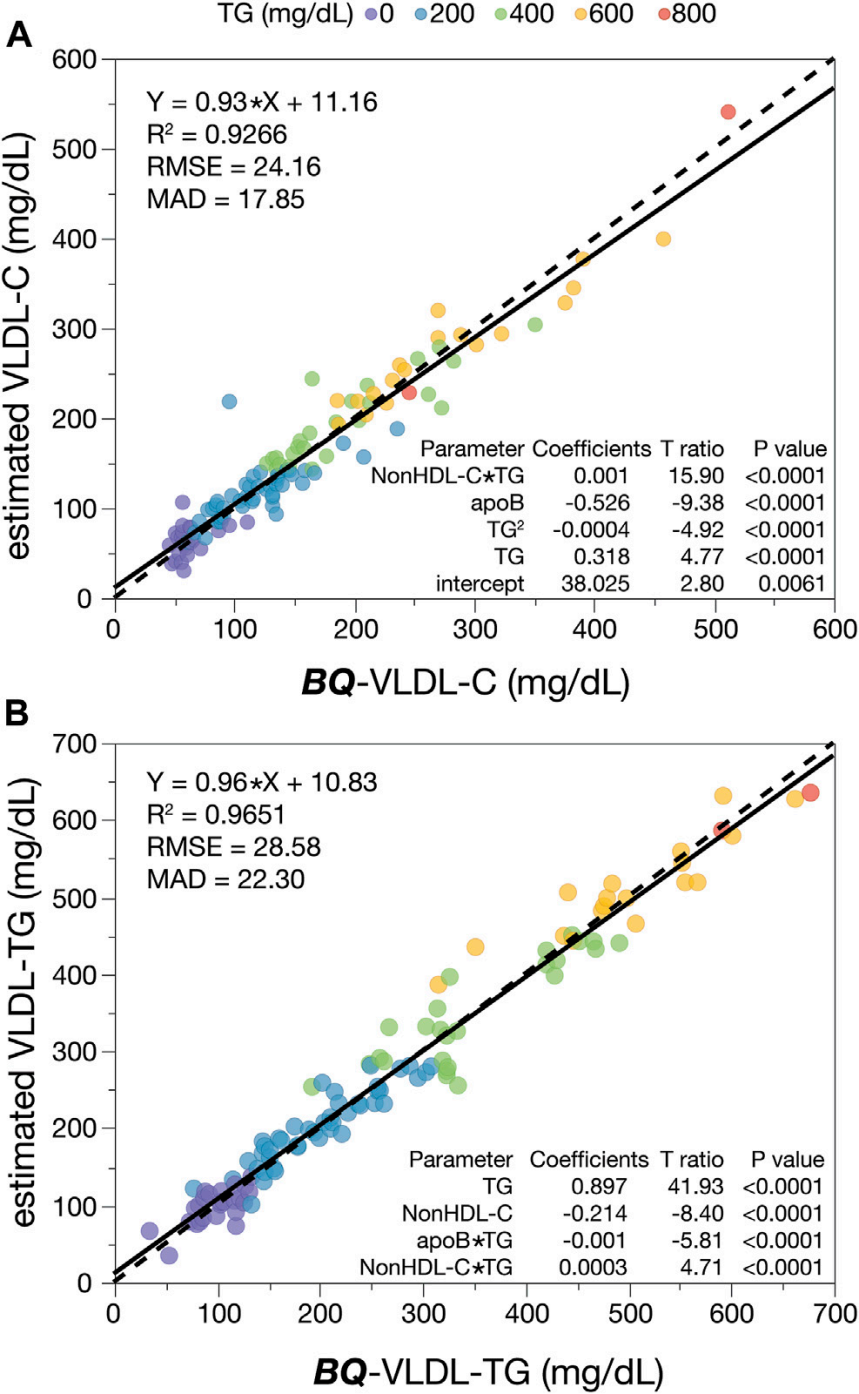
HLP3 specific equations for VLDL-C and VLDL-TG. HLP3 patients (N = 115) were used to develop a regression analysis for VLDL-C **(A)** and VLDL-TG **(B)** with the indicated input variables that include apoB. Solid lines are the linear fit for the indicated regression equation. Dashed lines are the line of identity. Results are color-coded by TG levels with the value in the legend indicating the start of each interval.

Finally, given the residual bias observed in the ***eS***-VLDL-C equation (Figure 3), we also developed specific equations that utilize apoB for estimating both VLDL-C (Figure 6A) and VLDL-TG (Figure 6B), once a patient is identified as having HLP3. As will be discussed in the following, these equations may potentially be used for monitoring response to lipid-lowering therapy for HLP3 patients.

## Discussion

Identification of HLP3 is useful in the management of these patients because their risk for cardiovascular diseases (CVD) is greater than what would be expected based on their standard lipid profile. In particular, LDL-C is a poor risk marker in this type of dyslipidemia not only because of their elevated TG but also because of a much greater fraction of cholesterol carried on remnant particles, which are known to be particularly proatherogenic (Hopkins et al., 2014). In addition, although these patients frequently show a good response to statins and lifestyle changes, which diminishes the impact of secondary contributory factors for this disorder, they can sometimes still have residual risk from remnant lipoproteins after statin treatment and may require a second lipid-lowering agent for lowering plasma TG.

We describe here two new methods for identifying HLP3. The first simply involves using results from the standard lipid profile for estimating VLDL-C, which is the original Sampson-NIH equation 1 (***S***-VLDL-C) (Sampson et al., 2020) that is currently used by clinical laboratories together with the Sampson-NIH equation 2 for LDL-C calculation. This approach has not been previously used in the case of the ***F***-VLDL-C equation because it always assumes a fixed ratio of VLDL-C/TG of 0.2. Although our new method based on the standard lipid panel shows a reasonably good sensitivity of over 70% for detecting HLP3, its specificity of about 80% is not ideal for a relatively rare disorder like HLP3. It may, however, have some utility as a screening test, because a positive presumptive score by this method could trigger the performance of a physical exam to look for signs and symptoms of HLP3. Results from this test could also be used by clinical laboratory information systems to automatically reflex to measuring apoB in order to make a more definitive diagnosis. We recently reported on a simple algorithm for using plasma non-HDL-C and TG for classifying all the common lipoprotein dyslipidemic phenotypes except for HLP3 and described how it can be used as a practical aid in the clinical management of patients (Sampson et al., 2021). With the use of the new method described here for HLP3, one can now identify all the common lipoprotein phenotype disorders from the standard lipid panel and the phenotypes could potentially be automatically reported along with the lipid test results.

The second method we developed for identifying HLP3 is both more sensitive and specific than the ***S***-VLDL-C equation, the ***F***-VLDL-C formula, or the dual lipid apoB ratio test. By using apoB, we were able to modify the ***S***-VLDL-C equation for improving its accuracy for VLDL-C. ApoB is present as a single copy per LDL (Knott et al., 1986), VLDL (Elovson et al., 1988), and chylomicron (Phillips et al., 1997) particle and thus provides a measure of the particle number for these lipoproteins. All of these lipoprotein particles are highly diverse in their size, which affects their TG and cholesterol carrying capacity. This likely accounts for why the inclusion of apoB in the ***eS***-VLDL-C equation improves its accuracy, particularly for HLP3, which is characterized by having a large number of small VLDL particles (Hopkins et al., 2014). Both terms in the ***eS***-VLDL-C equation that includes apoB have negative coefficients, which indicates that the use of apoB helps compensate for the overestimation of VLDL-C by the other variables in the equation. It is also worth noting that although apoB was not markedly elevated in HLP3 like it has been described for familial combined hyperlipidemia (Sniderman et al., 2018), it was modestly increased in at least some subjects. It is likely given the known metabolic defect in HLP3 that some of the excess apoB in HLP3 would reside in small dense VLDL rather than LDL, but this was not directly tested in this study and would be a valuable future research direction.

We also describe two other equations, which are specific for calculating the cholesterol and TG content of VLDL for already diagnosed HLP3 patients. These equations cannot be used for initially identifying HLP3 and should also not be used for normolipidemic patients because it will lead to an overestimation of VLDL-C and VLDL-TG. Nevertheless, these two equations may be potentially useful for monitoring response to lipid-lowering therapy for HLP3, but this will require additional future studies to determine if this is valuable or not.

A limitation of our approach is that an additional test, namely, apoB, will be required to more definitively make the diagnosis of HLP3. It has recently been shown, however, in several large studies that apoB is the most accurate lipid-related measure of CVD risk (Sniderman et al., 2019; Marston et al., 2022), and thus it will likely be used more often in the future even for primary screening. The 2019-European Society of Cardiology/European Atherosclerosis Society guidelines for the management of dyslipidemias (Mach et al., 2019), recommended that physicians consider measuring apoB for CVD risk assessment, particularly in patients with HTG, diabetes, obesity, metabolic syndrome, or low levels of LDL-C. These guidelines also state that when available, apoB can also be used as an alternative to LDL-C as the primary means to screen, diagnose, and manage patients.

Another limitation of our study is that we only compared the ***S***-VLDL-C equation (the original Samspon-NIH equation 1) and the ***eS***-VLDL-C equation to the ***F***-VLDL-C formula and did not examine other potential LDL-C equations. Because the original Sampson-NIH equation was previously shown to be more accurate than all other LDL-C equations, particularly for high TG samples (Sampson et al., 2020), it is unlikely that any alternative equation would be superior for identifying HLP3. In addition, none of the previously developed LDL-C equations, including the original Sampson-NIH equation, contained apoB as an independent variable like the ***eS***-VLDL-C equation. We also did not directly assess the clinical utility of the new ***eS***-VLDL-C equation in the routine clinical laboratory setting, which will have to await a future study. It will also be important to test the new ***eS***-VLDL-C equation in a wide variety of different ethnic/racial groups to determine its generalizability.

In summary, the two new equations, ***S***-VLDL-C and ***eS***-VLDL-C, that we describe for identifying HLP3 potentially have great practical value for the clinical management of this disorder because they only involve the use of the standard lipid panel and apoB, which are both relatively inexpensive and widely available. Future studies, however, will be needed in different types of patient cohorts to assess the clinical impact of early detection and monitoring of HLP3 patients by these equations.

## Data Availability

The raw data supporting the conclusions of this article will be made available by the authors, without undue reservation.

## Abbreviations

apoB, apolipoprotein B; ASCVD, atherosclerotic cardiovascular diseases; ß-VLDL, beta-migrating very low-density lipoproteins; BQ-VLDL-C, beta-quantification of VLDL-C; eS-VLDL-C, enhanced Sampson equation for VLDL-C; F-VLDL-C, Friedewald formula for VLDL-C; HLP3, hyperlipoproteinemia type III/dysbetalipoproteinemia; HTG, hypertriglyceridemia; S-VLDL-C, original Sampson equation for VLDL-C, the Sampson-NIH equation 1; VLDL-C, very low-density lipoprotein-cholesterol.
